# Origins of asexuality in *Bryobia *mites (Acari: Tetranychidae)

**DOI:** 10.1186/1471-2148-8-153

**Published:** 2008-05-19

**Authors:** Vera ID Ros, Johannes AJ Breeuwer, Steph BJ Menken

**Affiliations:** 1 Evolutionary Biology, Institute for Biodiversity and Ecosystem Dynamics, University of Amsterdam, P.O. Box 94062, 1090 GB Amsterdam, The Netherlands

## Abstract

**Background:**

Obligate asexual reproduction is rare in the animal kingdom. Generally, asexuals are considered evolutionary dead ends that are unable to radiate. The phytophagous mite genus *Bryobia *contains a large number of asexual species. In this study, we investigate the origin and evolution of asexuality using samples from 111 populations in Europe, South Africa and the United States, belonging to eleven *Bryobia *species. We also examine intraspecific clonal diversity for one species, *B. kissophila*, by genotyping individuals from 61 different populations. Knowledge on the origin of asexuality and on clonal diversity can contribute to our understanding of the paradox of sex.

**Results:**

The majority (94%) of 111 sampled populations reproduces asexually. Analysis of part of nuclear 28S rDNA shows that these asexuals do not form a monophyletic clade. Analysis of the mitochondrial COI region shows that intraspecific variation is extensive (up to 8.8%). Within *B. kissophila*, distinct clades are found, which are absent at the nuclear 28S rDNA level. Moreover, paraphyletic patterns are found at the mitochondrial DNA.

**Conclusion:**

Asexuality is widespread in the genus *Bryobia*, signifying that some animal taxa do contain a high number of asexuals. We argue that asexuality originated multiple times within *Bryobia*. *Wolbachia *bacteria cause asexuality in at least two *Bryobia *species and may have infected different species independently. The high intraspecific clonal diversity and the patterns of paraphyly at the mitochondrial DNA in *B. kissophila *might be explained by a high mutation fixation rate and past hybridization events. Reproductive parasites like *Wolbachia *and *Cardinium *might influence these processes. We discuss the role these bacteria could play in the evolutionary success of asexual species.

## Background

Asexual taxa are found across the eukaryotic tree of life: many plant, fungal, and animal taxa contain asexual lineages. In most cases however, this asexuality is facultative: apparent asexual species do have sex now and then. *Obligate *asexuality is less widespread. In the animal kingdom less than 1% of all species reproduce strictly asexually [1]. The distribution of these asexuals is 'tippy': most asexuals are found as single branches on the tips of the tree, branching off from closely related sexual species [1,2]. Fully asexual taxa contain few species. Apparently, asexual species can survive in the short-term, but are doomed to extinction in the long-term.

Asexual reproduction has short-term advantages compared to sexual reproduction. In a sexual population females will produce both sons and daughters, but in an asexual population females will produce only daughters. An asexual population has therefore twice the growth rate of a sexual population (assuming a sexually reproducing population with an equal sex ratio and with males contributing nothing but gametes to the offspring). In other words, there is a two-fold cost of sexual reproduction [3,4]. Besides this two-fold cost, there are other costs related to sexual reproduction: costs of finding a mate, of sexually transmitted diseases or selfish genetic elements, or of the act of sex itself [5]. Despite these costs, sex is widespread (the 'paradox of sex'). General explanations for this paradox refer to the long-term disadvantages of asexual reproduction: asexuals are less able to adapt to novel environments and are exposed to accumulation of deleterious mutations (reviewed in [1,6-10]). This is why asexuals are considered short-lived evolutionary 'dead ends' with limited adaptive potential [1,11,12]. It also explains the sporadic and low-level phylogenetic distribution of obligate asexual lineages. An exception to this pattern are a few groups that have been reproducing exclusively asexually for a long evolutionary time, like the bdelloid rotifers [13], darwinulid ostracods ([14]; but see [15]), and oribatid mites [16,17].

The phytophagous spider mite genus *Bryobia *(Acari: Tetranychidae) contains both asexually (thelytokous) and sexually (arrhenotokous) reproducing species. The genus is poorly studied and phylogenetic relationships are unknown. Species are described on the basis of morphology and host plant associations [18,19]. However, suitable morphological characters are rare in these tiny mites and this severely limits identification. In addition, host plant associations are generally considered unsuitable as primary input for species identification [20]. Bolland et al. [21] list over 130 species names, but these are likely to include synonyms and overlapping species descriptions, as such descriptions are often based on morphological descriptions of quantitative characters (*e.g*., body size, number and length of setae) in single, locally occurring mites. Nonetheless, for the majority of described species, no males have been reported and females reproduce asexually through thelytokous parthenogenesis [22], indicating that asexuality is widespread in this genus.

Parthenogenesis in at least two asexual *Bryobia *species is caused by the bacterial endosymbiont *Wolbachia *[23]. *Wolbachia *are reproductive parasites that enhance their own transmission by manipulating the reproduction of their host, resulting in an increased number of infected females (see [24] for a review). *Wolbachia *were detected in four additional asexual *Bryobia *species, but the causal effect was not established [23]. In addition, Weeks and Breeuwer [23] showed that the parthenogenesis is functionally apomictic, as heterozygosity is maintained.

The occurrence of many asexuals in one genus is rare and raises questions about the origin and evolution of the asexual lineages. One way to address such questions is using a phylogenetic approach [25]. Suppose a phylogeny shows that asexuals occur as single lineages among sexual sister groups, indicating that they are 'evolutionary dead ends' that are unable to radiate. Then, the most likely explanation is multiple (and recent) origins of asexuality. On the contrary, if all asexuals form a monophyletic group, the most likely explanation would be a single and older origin with subsequent radiation of asexuals, a phenomenon that has rarely been found for asexuals [2].

In this study, we investigate the phylogenetic history of asexual reproduction in the genus *Bryobia*. Also, we examine intraspecific clonal variation by analyzing samples collected on a large geographic scale. Generally, clonal species are thought to harbor little genetic diversity. This approach provides the framework for investigating the evolution of asexuality and host plant specificity across the genus. We use a combination of mitochondrial (the cytochrome oxidase *c *subunit I gene, COI) and nuclear (the 28S rDNA gene) sequence data for inferring species relationships. Combining nuclear and mitochondrial data is desirable for detecting processes such as hybridization and hitchhiking.

## Methods

### Sampling and DNA extraction

We sampled *Bryobia *mites from 111 different locations (populations) between May 2000 and September 2006 (see Additional file 1 and 2 for details on *Bryobia *samples). This collection comprised samples from a wide range of host plant species (at least 12 different host plant genera in six families) in 14 different countries in Europe. We investigated intraspecific variation in one species (*B. kissophila*) by analyzing 61 populations across 12 different European countries. Additionally, we included samples from South Africa (one population) and the United States (two populations). As an outgroup reference, we sampled ten European and two Chinese *Petrobia *spp. (Acari: Tetranychidae) populations. *Bryobia *and *Petrobia *both belong to the subfamily Bryobiinae of the Tetranychidae [21]. Mites were either directly used or stored in 96% ethanol until DNA extraction. Mites were morphologically identified by Dr. F. Faraji (Mitox, Amsterdam) and Dr. E. Ueckerman (PPRI, Pretoria, South Africa). However, not all samples could be identified morphologically. Currently, few morphological keys for distinguishing *Bryobia *species exist, but none of these includes all described species and all are developed for identifying locally occurring mites only ([19,26] (South Africa); [27] (Greece); [28] (New Zealand); [29] (United States)). Voucher specimens will be stored at the Zoological Museum of the University of Amsterdam (ZMA). DNA was extracted from single mites using the CTAB extraction method as previously described [30]. Adult females as well as males, if present, were used.

### Species identification

Samples were identified to the species level using morphological keys. *Bryobia sarothamni *Geijskes, *B. rubrioculus *(Scheuten), *B. berlesei *van Eyndhoven, *B. praetiosa *Koch, and *B. kissophila *van Eyndhoven were identified based on morphology. Samples that could not be morphologically identified were *a posteriori *named *B*. spec. I-VII, based on molecular phylogenetic analysis. The latter designations will be maintained throughout the article, although the exact species status remains to be determined (see discussion). In addition, we sampled mites from the related genus *Petrobia*. These were identified as *Petrobia tunisea *and *P. harti*. Other *Petrobia *samples were *a posteriori *named *P*. spec. I and *P*. spec. II.

### (A)sexuality of species

Where possible, field-collected females were reared as isofemale lines in the laboratory. In this way the reproductive mode could be assessed. Strains, in which males were neither observed in the cultures, nor encountered in the field, were classified as asexual. Males are easily recognized by their smaller body size and extremely long front legs (up to two times their body size) compared to females, and by their mating behavior [22]. Additionally, the lower part of the abdomen is V-shaped in males, while it is circular in females. For those species that were not reared in the lab, the occurrence of males in the collected field samples was assessed (Table 1). If males were encountered, the clade was considered to reproduce sexually; if not, the clade was classified as asexual.

**Table 1:** Reproductive mode of *Bryobia *and *Petrobia *species.

Species	Reproductive mode	Method	Males present	N
*B. berlesei*	A	culture	No	
*B. kissophila*	A	culture	No	
*B. praetiosa*	A	culture	No	
*B. rubrioculus*	A	culture	No	
*B. sarothamni*	S	culture	No	
*B*. spec. I	A	culture	No	
*B*. spec. II	A	field	No	31
*B*. spec. III	A	field	No	16
*B*. spec. IV	S	field	Yes	12
*B*. spec. V	A	culture	No	
*B*. spec. VI	A	field	No	31
*B*. spec. VII	A	field	No	14
*P. harti*	S	field	Yes	58
*P. tunisea*	S	field	Yes	94
*P*. spec. I	A/S*	field	No	4
*P*. spec. II	A	culture	No	

A = asexual, S = sexual. Reproductive mode was assessed by determining the presence of males directly in field samples only (field), or in combination with laboratory cultures initiated with field samples (culture). N indicates the total number of individuals investigated (in cases where males were counted from field samples only). * = mode of reproduction is uncertain because of the small sample size.

### PCR amplification and sequencing

Part of the mitochondrial COI gene was amplified using various primer combinations (as individual primer sets did not work for all species) (Table 2). Two primers were adjusted based on sequences form other tetranychid taxa available from GenBank (Table 2). Depending on the primer combination, a fragment of 410–867 basepairs (bp) was obtained. A fragment of 410 bp, amplified by the primers COI_F1 and COI_R1 (Table 2; [31]), was used in subsequent analyses. PCR conditions for COI were as described in Ros and Breeuwer [30]. For one individual of each COI haplotype, the 5' end of the nuclear 28S rDNA (the D1 region) was amplified (see Additional file 1 and 2). This region is generally less variable than the mitochondrial COI region [32] and therefore, samples with identical COI haplotypes were assumed to have an identical 28S haplotype. However, several processes might violate this assumption (see discussion). The validity of the assumption was therefore investigated by sequencing more than one individual per COI haplotype in several cases (see Additional file 1 and 2). PCR reaction mix was the same as for COI [30], except that no additional MgCl_2 _was added. PCR cycling conditions for 28S were 2 min. at 94°C, followed by 35 cycles of 40 sec. at 94°C, 40 sec. at 48°C, and 90 sec. at 72°C, and a final extension at 72°C for 5 min. A negative control in which water was added instead of DNA was included in all PCR reactions. Products were visualized on a 1% agarose gel stained with ethidium bromide in 0.5× TBE buffer (45 mM Tris base, 45 mM boric acid, and 1 mM EDTA; pH 8.0).

**Table 2:** Primer sequences.

Primer	Sequence 5' to 3'	Reference	Position
COI_F1	TGATTTTTTGGTCACCCAGAAG	[31]	2173
COI_F2	AAGAGGAGGAGGAGACCCAA	[77]	2133
COI_F3	WGTHTTAGCAGGAGCAATTACWAT	modified from [78,79]	2067
COI_F4	GGAGGATTTGGAAATTGATTAGTTCC	[80]	1693
COI_R1	TACAGCTCCTATAGATAAAAC	[31]	2605
COI_R2	AAWCCTCTAAAAATRGCRAATACRGC	modified from [77]	2620
28S_D1_F	ACCCSCTGAAYTTAAGCAT	[81]	
28S_D1_R	AACTCTCTCMTTCARAGTTC	[81]	

Primers used for amplification and sequencing of part of the COI and 28S D1 region. The position of the COI primers on the COI gene is listed, position numbers correspond to the *Drosophila melanogaster* mitochondrial DNA sequence [GenBank:U37541]. The fragment between primers F1 and R1 (410 bp) was used for analyses in this study.

PCR products were purified using a DNA extraction kit (Fermentas, St. Leon-Rot, Germany). The purified products were directly sequenced using the ABI PRISM BigDye Terminator Sequence Kit (Applied Biosystems, Nieuwerkerk a/d IJssel, The Netherlands) according to the manufacturer's instructions but diluted 16 times. Both strands of the products were sequenced using the same primers as in the PCR amplification. Sequences were run on an ABI 3700 automated DNA sequencer. Sequences were checked visually for ambiguous nucleotides (double peaks) and the presence of stop codons. Sequences of representatives of all haplotypes were submitted to GenBank (see Additional file 1 and 2 for GenBank accession numbers).

### Data analysis

Sequences were aligned using ClustalX version 1.8.0 with default settings [33]. Analyses were performed for the 28S and COI datasets separately. PAUP* version 4.0b10 [34] and DAMBE version 4.1.15 [35] were used to calculate numbers of variable sites, uncorrected pairwise divergences, nucleotide composition, and transition and transversion ratios. PAUP was used to perform a chi-square test of base frequency homogeneity across all taxa.

Phylogenetic analyses were performed using Neighbour-Joining (NJ), Maximum Likelihood (ML), and Bayesian methods. Both PAUP and Modeltest 3.6 [36] were used to select the optimal evolution model. The selected model was further optimized by critically evaluating the selected parameters [37] using the Akaike Information Criterion (AIC; [38]). For 28S, a submodel of the GTR+G (General Time Reversible model with gamma distributed rate heterogeneity among sites) with a rateclass 'a b c a b d' had the highest likelihood score (i.e., the lowest -ln likelihood score) (AIC). Because COI is a protein coding gene, we tested if the likelihood of models could be further improved by incorporating specific rates for each codon position [39]. Using this approach, the Transition model (TIM) with site specific rates for the three codon position was selected for COI. Under the selected models, parameters and tree topology were optimized using the successive approximations approach [40]. NJ analyses (p-distances) and ML analyses (heuristic search, random addition of sequences with five replicates, TBR branch swapping, and a reconnection limit of 10 for COI analysis) were performed in PAUP. Robustness of nodes was assessed with 1,000 NJ- resp ML-bootstrap replicates. However, as PAUP does not allow for site-specific rates in bootstrap analysis, ML bootstrapping for COI was performed with gamma distributed rates, with 100 bootstrap replicates. Bootstrap values were then plotted on the phylogeny obtained with the TIM model with site specific rates. Bayesian analyses were performed as implemented in MrBayes 3.1.2 [41]. For 28S we used a GTR+G model; for COI we used a GTR model with separate rates for each codon position. Analyses were initiated form random starting trees. Two separate MCMC (Markov chain Monte Carlo) runs, each composed of four chains (one cold and three heated), were run for 2 million generations. The cold chain was sampled every 100 generations, the first 5,000 generations were discarded afterwards (burn-in of 25%). Posterior probabilities were computed from the remaining trees. We checked whether the MCMC analyses ran long enough using the program AWTY (Are We There Yet?) [42]. Stationarity was assumed when there was convergence between the two MCMC runs and when the cumulative posterior probabilities of splits stabilized; in both analyses 2 million generations proofed sufficient. The final trees were rooted using four species of the genus *Petrobia *as outgroup.

### Test of monophyly

The COI phylogeny depicts *B. kissophila *as a paraphyletic species (see results). We tested if a phylogeny with *B. kissophila *as a monophyletic group is significantly less likely than the presented phylogeny by performing a Kishino-Hasegawa test (KH-test; [43]) and a Shimodaira-Hasegawa test (SH-test; [44]) as implemented in PAUP. First, we performed a constrained heuristic search (*B. kissophila *as a monophyletic clade; search settings same as without constraint). The KH- and SH-test were used to test for significant differences between the likelihood scores of the trees (unconstrained and constrained). We used a one-sided KH-test to correct for comparing an *a priori*-specified phylogeny (the constrained tree) with an *a posteriori*-specified phylogeny (the ML tree) [45]. We also tested for monophyly of all asexual respectively sexual species using the same approach (constrained search with asexuals resp. sexuals as a monophyletic clade).

## Results

### Patterns of molecular evolution

#### The 28S D1 fragment

**Figure 1:**
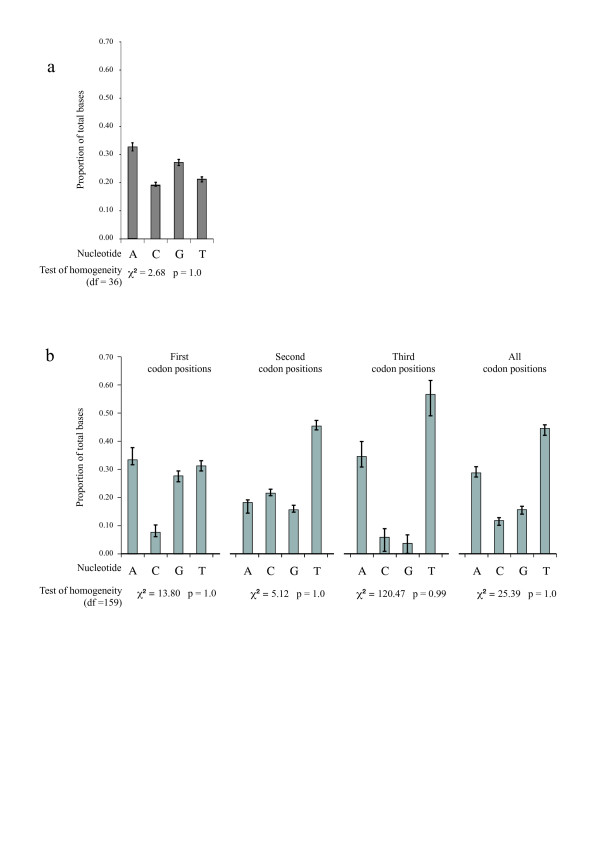
**Base compositions**. Base composition, averaged over all *Bryobia *samples, of a) the 28S region and b) the COI region. Error bars depict minimum and maximum values. Results of the homogeneity test are given below the graphs. For the COI region, data are shown for each codon position separately and for the three positions together.

**Figure 2:**
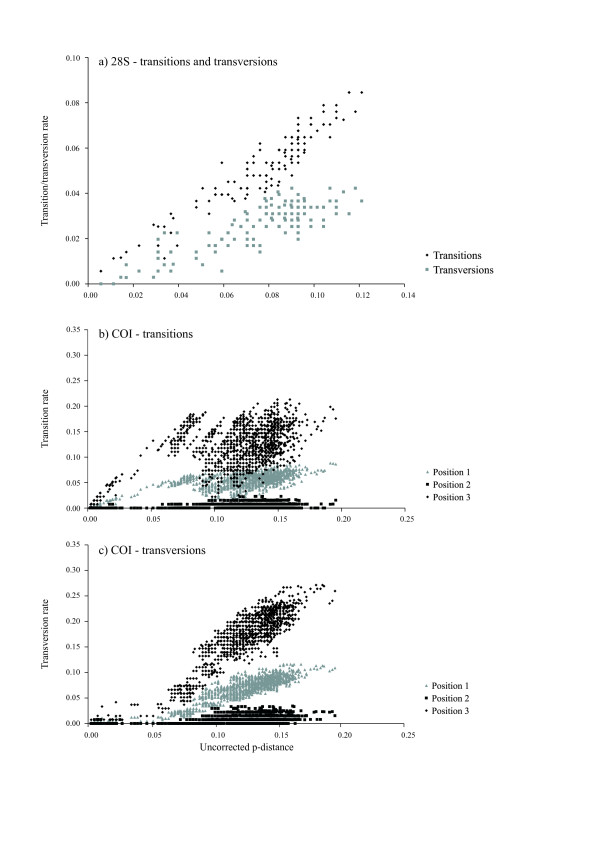
**Saturation plots**. Saturation plots of a) transition and transversion rates against uncorrected p-distance for the 28S region and b) transition and c) transversion rates against uncorrected p-distance for each codon position separately for the COI region.

The 28S D1 fragment could be amplified in all but one *Bryobia *species (amplification in *B*. spec. II failed). No signs of ambiguity were found and intraspecific variation was absent or very low (0–1%), indicating that intra-individual variation is absent for 28S D1 (which is a multiple copy gene). Therefore, it was decided not to clone the PCR products before sequencing. The length of the amplified 28S fragment was 356 bp. This included the D1 region (168 bp) and its flanking regions (79 bp before and 109 bp after the D1 region) [46]. Of the 356 sites, 60 sites were phylogenetically informative, 20 sites variable but uninformative, and 276 sites were constant. In two species (*B. sarothamni *and *B*. spec. VI) an insert of one bp was found. All sequences could be unambiguously aligned. On average, across all *Bryobia *taxa, the AT content was 54% (33% A, 21% T, 19% C, and 27% G), i.e., an unbiased basepair composition. A chi-square test of base frequency homogeneity revealed no significant differences in basepair composition across taxa (Figure 1a). The extent of saturation was assessed by plotting the transition and transversion rates against the uncorrected p-distance divergences (Figure 2a). Transitions outnumbered transversions as the divergence time increased and both did not reach a plateau. This observation, plus the fact that the basepair composition was unbiased, indicates that saturation is absent and that this fragment can be used for phylogeny reconstruction.

#### The COI fragment

The length of the amplified COI region ranged from 410 to 867 bp (depending on the choice of primer combinations); a homologous 410 bp fragment (Table 2) was used for subsequent analyses. All sequences could be unambiguously aligned; no insertions or deletions were found. Translation of the sequences into amino acids revealed no stop codons. Of the 410 sites, 142 sites were phylogenetically informative, 10 sites were variable but uninformative, and 258 sites were constant. On average, across all *Bryobia *taxa, the AT content was 73% (29% A, 44% T, 12% C, and 15% G). This AT bias is comparable to that found for other Tetranychidae [30] or for insects [47]. However, the bias in base composition was not uniformly distributed over codon positions (Figure 1b). First, second, and third codon positions showed AT biases of 65, 63, and 91%, respectively. In some haplotypes, the G base was entirely absent at the third codon position. A chi-square test of base frequency homogeneity revealed no significant differences across taxa for the overall data set or for the three base positions separately (Figure 1b). Transversions outnumbered transitions as the divergence time increased, predominantly at the third codon position (Figure 2b and 2c). This indicates saturation, although the transversion rate did not reach a plateau yet. The extremely biased base composition combined with saturation at the third codon position severely limits the use of this COI region for resolving phylogenetic relationships, especially at deeper nodes. Exclusion of the third codon position did not result in a better resolution because variation at the first and second codon position was very low. A high AT content and the resulting limited phylogenetic resolution for the COI region was also found for other tetranychid mites [30] as well as for parasitengona mites [48] and velvet worms (Onychophora) [49].

### 28S phylogeny

**Figure 3:**
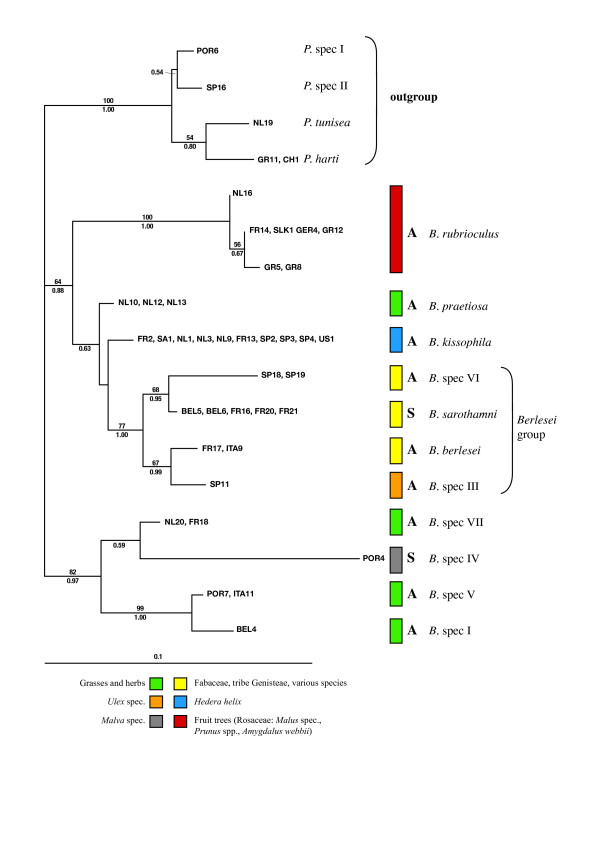
**28S rDNA tree**. Maximum likelihood tree of the genus *Bryobia *and four outgroup species of the genus *Petrobia *for the nuclear rDNA 28S D1 region. For each haplotype (branch tip) samples sharing that haplotype are given (see Additional file 1 and 2 for abbreviations and sample details). Clades are colored according to their host plant association (see legend at the bottom). Clades are followed by the mode of reproduction (A = asexual, S = sexual) and presumed species name. Numbers above branches indicate the percentage bootstrap values based on 1,000 replicates, numbers below branches depict Bayesian posterior probabilities (only values larger than 50 (ML) and 0.50 (Bayesian) are indicated). The bar at the bottom indicates a branch length of 10% likelihood distance.

Figure 3 shows the ML phylogeny reconstructed from the 28S D1 fragment, with ML bootstrap values and Bayesian posterior probabilities. Identical topologies were obtained from the different analyses (NJ, ML and Bayesian). The resulting phylogeny is well-resolved. Populations belonging to a single lineage have different geographic origins and are invariably found on the same host plant species or group of host plant species. This indicates that each species has a strong host plant association. Certain species group together, and this grouping is again linked to related host plant species. *Bryobia berlesei*, *B. sarothamni*, *B*. spec. III, and *B*. spec. VI form a monophyletic group (bootstrap value 77%) and are all found on host plant species of the Fabaceae, tribe Genisteae. *Bryobia *species found on host plant species of the tribe Genisteae have been named the Berlesei group [18]. Closely related to this group are *B. kissophila *and *B. praetiosa*, although exact relationships remain unresolved (low bootstrap values). *Bryobia kissophila *is restricted to *Hedera helix *(Ivy), whereas *B. praetiosa *is found on grasses and herbaceous plant species growing along road sides. Samples from rosaceous fruit tree species also form a monophyletic group (bootstrap value 100%). Here, three haplotypes are distinguished. The p-distances between these haplotypes are low (0.6–1.0%) and these haplotypes most likely all concern *B. rubrioculus*. All aforementioned species group together (bootstrap value 64%) and are clearly separated from another group of species collected from grasses and herbs (*B*. spec. I, *B*. spec. V, and *B*. spec. VII) and *Malva *spec. (*B*. spec. IV). Average distances between *Bryobia *species range from 1.7 to 11.5%.

### COI phylogeny

**Figure 4:**
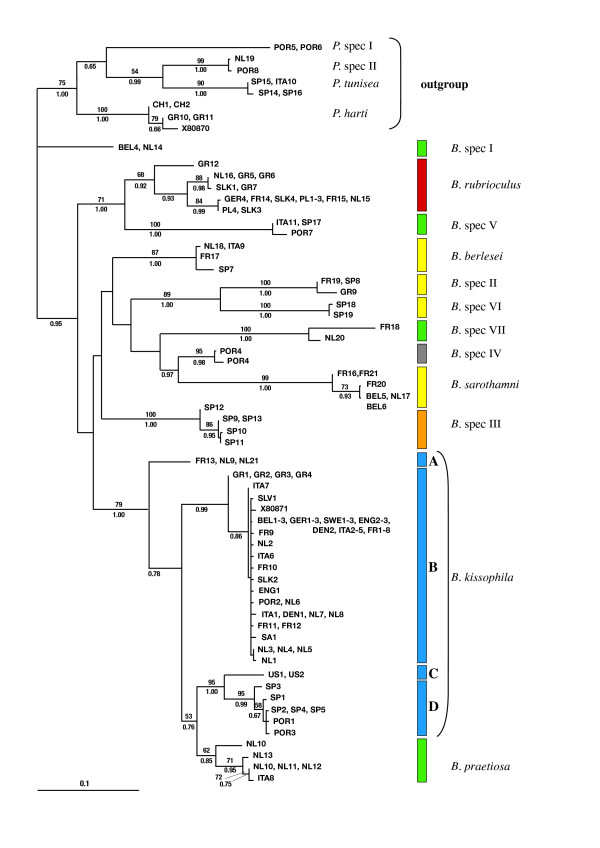
**COI tree**. Maximum likelihood tree of the genus *Bryobia *and four outgroup species of the genus *Petrobia *for part of the mitochondrial COI region. For each haplotype (branch tip) samples sharing that haplotype are given (see Additional file 1 and 2 for abbreviations and sample details). Clades are colored according to their host plant association (see legend in Figure 3). Clades are followed by their presumed species name. Clades of *B. kissophila *are coded as A-D. Numbers above the branches indicate the percentage bootstrap values based on 100 replicates, numbers below branches depict Bayesian posterior probabilities (only values larger than 50 (ML) and 0.50 (Bayesian) are indicated). The bar at the bottom indicates a branch length of 10% likelihood distance.

Figure 4 shows the ML phylogeny reconstructed from the COI fragment, with ML bootstrap values and Bayesian posterior probabilities. NJ, ML, and Bayesian analyses show identical topologies. The COI region is more variable than the 28S D1 region, with interspecific distances ranging from 7.5 to 16.8%. The ML phylogeny reconstructed from COI sequences shows clustering of haplotypes into distinct clades (Figure 4), which in most cases correspond to the 28S lineages found. Clades found for COI are well supported; however, the phylogenetic relationships between these clades remain largely unresolved. Therefore, higher-level groupings (*e.g*., the Berlesei group) are less pronounced than for 28S. The wide sampling of *B. kissophila *revealed a large amount of intraspecific variation. Samples from 61 populations cluster into four different clades (named A-D in Figure 4). Average pairwise distances between these clades range from 5.5 to 8.8%. Clade B is the largest, covering samples from all over Europe. Samples of clade A fall within the geographical range of clade B (clade A contains one French and two Dutch populations). Clade C is comprised of populations from the United States and clade D of populations from Spain and Portugal (note however that the sampling intensity in clade C is low).

Based on the COI sequences, *B. kissophila *is paraphyletic; *B. praetiosa *forms a monophyletic group, which is nested within the *B. kissophila *clades. The same topology is supported by the NJ and Bayesian analysis (data not shown). However, the KH-test and SH-test do not reject a topology with a monophyletic *B. kissophila *clade (p = 0.51 for both tests), so paraphyly of *B. kissophila *is not well supported. All *B. kissophila *COI clades share the same 28S haplotype, which differs 1.7% from *B. praetiosa*. Nucleotide differences between *B. praetiosa *and the four *B. kissophila *clades range from 5.1 to 5.9% and are thus smaller than those among *B. kissophila *clades (5.5 to 8.8%).

For all species except *B. rubrioculus*, all COI haplotypes found within the various species clades share an identical 28S haplotype. The assumption that samples with identical COI haplotypes have an identical 28S haplotype proved also to be true in cases where this was tested. In *B. rubrioculus*, however, this assumption was violated, as two samples sharing the same COI haplotype (NL16 and GR5) have different 28S haplotypes (distances between 0.6 and 1.0%).

### Phylogenetic distribution of asexuality

Despite extensive sampling, from different host plant species in various countries, only seven out of 111 sampled populations contained males (6%). These samples concern two species: *B*. spec. IV and *B. sarothamni*. All other populations reproduced asexually, indicating that asexuality is widespread in the genus *Bryobia *(Table 1). In the 28S phylogeny, the asexual species do not form a monophyletic clade. This is supported by the KH- and SH-test: a phylogeny enforcing asexuals as a monophyletic clade is significantly different from the maximum likelihood phylogeny (p = 0.002 for both tests). The two sexual species form two apical branches within different asexual clades. This pattern does not support the general observation that each asexual species forms a single apical branch with a sexual sister species. The observed patterns indicate either a single origin of asexuality with subsequent reversals to sexuality or multiple origins of asexuality. Unfortunately, the observed patterns in the 28S phylogeny could not be confirmed by the COI phylogeny, which is unresolved at deeper branching patterns due to saturation.

## Discussion

We present a detailed study of the genus *Bryobia*, using samples collected from a wide range of host plants across Europe. Our phylogenetic approach shows several distinct clades or lineages, supported by both nuclear 28S and mitochondrial COI data. For those species that could be morphologically identified, these clades are consistent with morphological data. Other clades are tentatively named *B*. spec. I-VII. Except for *B*. spec. IV these species all reproduce asexually. The assessment of species in asexual taxa is problematic, as the biological species concept is not applicable and each clonal lineage can be considered as a single species. However, Fontaneto et al. [50] showed that even in ancient asexual bdelloid rotifers, well-separated genetic clusters are found. In the genus *Bryobia *we find distinct phylogenetic entities, here tentatively labeled with different species names. Most species are restricted to a single host plant species (Figure 3 and 4). *Bryobia kissophila*, *B*. spec. III, *B. berlesei*, *B. sarothamni*, and *B*. spec. IV are each restricted to a specific host plant species and are thus true specialists. Furthermore, *B. rubrioculus *is restricted to rosaceous fruit tree species. However, different species may co-occur on a single host plant species. For example, both *B. sarothamni *and *B. berlesei *are found on common broom (*Cytisus scoparius*). Also *B*. spec II and *B*. spec VI are found on broom-like species, although they might each be restricted to a different broom species [18]. Finally, four species (*B. praetiosa*, *B*. spec. I, spec. V, and *B*. spec. VII) are more generalistic, feeding on several host plant species belonging to different families. The high level of specificity found for most *Bryobia *species contrasts with findings of Groot [51], who showed that three asexual species of *Brevipalpus *mites were host plant generalists, found on over 30 different host plant species with several host plant species shared among species. With an intensive sampling effort, including samples form a wide geographic area and a variety of host plants, we sampled 12 *Bryobia *species. This is in contrast with the total number of described species (see compilation by Bolland et al. [21]). We think that the number of species listed might be an overestimation due to the general lack of informative morphological characters for reliable species identification and the existence of synonymous species names – similar individuals collected from different geographic areas or host plants have been given different species names.

Below, we will first discuss the high levels of intraspecific clonal diversity found for *B. kissophila *and subsequently we will focus on the origin and evolution of asexuality within the genus *Bryobia*.

### Clonal diversity

Extensive sampling of *B. kissophila *shows that intraspecific diversity at COI is very large. Four clades (A-D) are distinguished, with interclade distances ranging from 5.5 to 8.8%. Clade (B) is the largest clade and comprises populations from all over Europe. Two other clades (C and D) are linked to geographically different areas (United States and Iberian Peninsula, respectively). The fourth clade (A) contains three populations from areas within clade B.

Intraspecific COI differences between some *B. kissophila *clades are larger than interspecific distances between *B. kissophila *and *B. praetiosa*. In the phylogenetic tree (NJ, ML, and Bayesian analyses) *B. kissophila*, which is only found on *Hedera helix*, forms a paraphyletic species: *B. praetiosa *falls within the *B. kissophila *clade. However, bootstrap support is not very high and a KH- and SH-tests do not exclude monophyly of *B. kissophila*. Still, the overlapping range of inter- and intraspecific divergence is remarkable. Intraspecific differences are much higher than values found in for example asexual *Brevipalpus *mites (Acari: Tenuipalpidae). In this mite genus intraspecific ML distances range between 1.9 to 4.3% for the same COI region [51]. Extremely high intraspecific differences (up to 20.9%) were found between sexual and asexual lineages of the ostracod *Eucypris virens *[52].

There are several hypotheses that could explain this high level of intraspecific diversity in asexual species. Clonal diversity can originate in at least three different ways [53]: 1) through separate and recurrent origin of clones from a sexual ancestor, 2) through hybridization between asexual females with males (either from the same or from other species), or 3) through mutation. Separate origins of clones from a sexual ancestor is often found in species with mixed reproduction (sexual as well as asexual lineages), where asexual clones are continuously formed over time [52]. Mixed reproduction is absent in *B. kissophila*, because the whole species is asexual. It is still possible that the different asexual clones independently originated from a highly variable sexual ancestor [54] and that ancestral mitochondrial polymorphism is maintained. This would, however, result in a correlation between the nuclear and mitochondrial phylogeny. The fact that the distinction between *B. kissophila *and *B. praetiosa *(1.7% difference) at the nuclear 28S locus is not supported by the mitochondrial COI phylogeny is not consistent with the above expectation.

Hybridization as a current source of clonal diversity, is not very likely because males are completely absent in *B. kissophila *as well as in *B. praetiosa*. Males have never been observed in cultures or in the field. Moreover, Weeks and Breeuwer [23] showed that males obtained after antibiotic treatment do not successfully mate with females. Furthermore, although males do exist in other species, these species are restricted to very different host plant species. On the other hand, hybridization might have occurred in the past before the origin or fixation of asexuality. Then, the phylogenetic pattern we currently observe is simply a reflection of the relatively recent fixation of asexual reproduction in different lineages.

Finally, mutations could explain part of the clonal diversity. We observed high levels of intraspecific diversity at the mitochondrial level, but almost none at the nuclear level. High levels of intraspecific mitochondrial COI diversity either signify a long history of asexuality or an elevated rate of mutations at the mitochondrial DNA. In the case of a long asexual history, divergence at nuclear genes is also expected, and alleles within an individual would eventually diverge over time (Meselson effect; [13]). However, if gene conversion occurs during meiosis, divergence at nuclear genes can be reduced or absent. Although heterozygosity is maintained in *Bryobia*, indicating that parthenogenesis is functionally apomictic [23], the mechanism of parthenogenesis is unknown. Maintenance of heterozygosity can be achieved by strictly apomictic parthenogenesis (no meiosis) or by premeiotic doubling. Only in the latter case, meiosis is present, and gene conversion can take place. It is unclear whether parthenogenesis in *Bryobia *involves meiosis, and at this moment we can not distinguish between the alternative explanations.

Reproductive parasites like *Wolbachia *and *Cardinium *can influence processes like hybridization and fixation of mutations and can therefore have a large impact on clonal diversity. They can cause selective sweeps of mitochondrial DNA, which increase the rate of fixation of mutations. Selective sweeps can either decrease (homogenization of mitochondrial variation in a population) or increase (when a species is infected with different bacterial strains each linked to a mitochondrial haplotype) genetic variation [55]. The pattern we found for *B. kissophila*, with distinct mitochondrial clades with high interclade divergence, was also found in *Drosophila simulans *[56,57]. In *D. simulans *these mitochondrial clades were most likely associated with different *Wolbachia *strains. Selective sweeps combined with hybridization between different species can also cause homogenization of species after a hybridization event, when the parasites spread and drag along the associated mitochondrial haplotype. This can result in paraphyletic patterns at the mitochondrial DNA. Such mitochondrial introgression patterns have indeed been found in several closely related species of *Drosophila *[58,59] and in species of the blowfly genus *Protocalliphora *[60]. *Wolbachia *are present in both *B. kissophila *and *B. praetiosa *[23]. It is possible that the different haplotypes observed within *B. kissophila *are a consequence of selective sweeps caused by different *Wolbachia *strains. Additionally, interspecific transfer of mitochondrial DNA from *B. kissophila *to *B. praetiosa *could also explain the paraphyly observed for *B. kissophila *and *B. praetiosa*.

In conclusion, both mutations and hybridization can explain the clonal diversity and paraphyletic patterns in *B. kissophila *and *B. praetiosa *and both processes are possibly driven by reproductive parasites. These processes might cause similar divergence patterns in other *Bryobia *species and explain incongruencies between 28S and COI phylogenies for *B. rubrioculus *as well.

The observed high levels of intraspecific variation at the COI gene have serious consequences for the use of COI as a tool for identifying species (DNA barcoding; [61,62]). Although the region we investigated is a different COI fragment than is usually used in barcoding studies (the Folmer fragment; [63]), a similar pattern may be expected for the total COI gene [30]. DNA barcoding assumes that intraspecific variation is lower than interspecific variation. A standard threshold of 2% divergence for identifying species has been proposed [61]. With intensive intraspecific sampling over a wide geographic range we demonstrated that intraspecific variation can be extensive and easily exceeds interspecific differences, thus undermining current barcoding assumptions. Similar high levels of intraspecific variation are not restricted to *Bryobia *mites but were also observed in other tetranychid mites [30].

### Origin of asexuality

**Figure 5:**
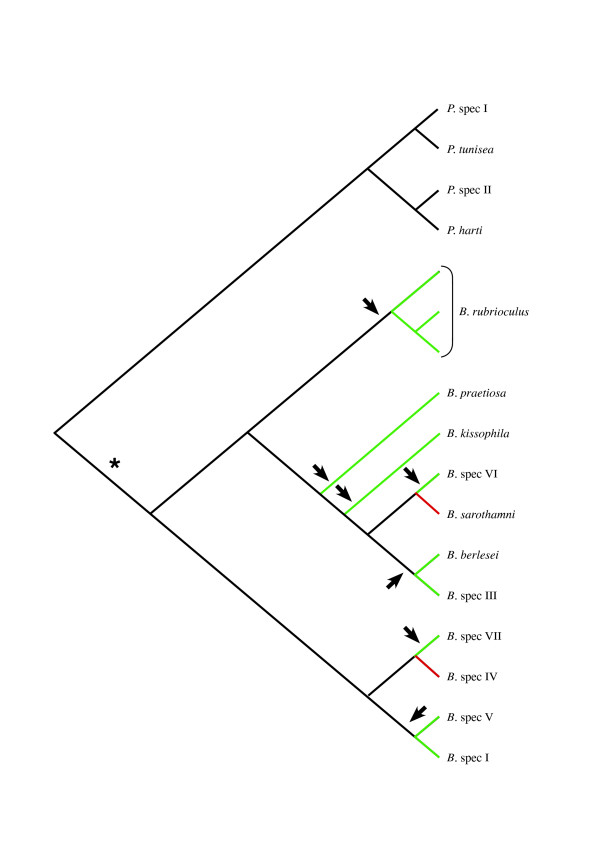
**Origins of asexuality**. Cladogram of the 28S tree of Figure 3 showing the relationships between asexual and sexual *Bryobia *species. Green and red branches indicate asexual and sexual species respectively. Black arrows indicate the minimum number of times asexuality has originated (note that the actual origin of asexuality might occur anywhere along the indicated branch). A single origin of asexuality at * requires at east two reversals to sexuality (red branches).

The 28S phylogeny shows that the asexual *Bryobia *species do not form a monophyletic clade (Figure 5). This implies two possible scenarios for the origin of asexuality in the genus: 1) asexuality originated once, with subsequent radiation of asexuals and at least two independent reversals to sexuality or 2) asexuality originated multiple times (at least seven times; Figure 5). The first scenario is the most parsimonious, because only three transitions (one from sexual to asexual, and two from asexual to sexual) are needed instead of seven (sexual to asexual) to explain the observed phylogenetic pattern. However, there are several arguments against this scenario.

First, reversals to sexuality are less likely than origins of asexuality. Once a species becomes strictly asexual, mutations are expected to accumulate in traits involved in sexual reproduction [2,64,65] and the capacity to produce sexually is lost very quickly. This process will also operate in species where asexuality is caused by bacteria. In species with bacterial-induced asexuality, including *B. praetiosa*, experiments have shown that females fail to reproduce sexually after removal of the bacteria [23,51,66-68]. Moreover, Groot and Breeuwer [69] showed that in *Brevipalpus *mites where *Cardinium *causes asexual reproduction, some strains lost the *Cardinium *bacteria but still reproduced asexually. Apparently, loss of *Cardinium *did not result in a reversal to sexuality. Although we can not exclude the possibility of reversal to sexuality [25], the aforementioned studies suggest that it is not likely.

Second, although we performed an extensive geographic sampling of species, sampling of the genus is not complete. For example, van Eyndhoven [18] described thirteen species as members of the 'Berlesei' group, three of which are sexual. We have only sampled four species of this group, one of which reproduces sexually. If more sexual species belong to this group, we need to invoke more reversals to sexuality in order to maintain the hypothesis that asexuality has a single origin in *Bryobia *(Figure 5).

Third, several independent infections with *Wolbachia *could explain independent origins of asexuality. Asexuality in *B. praetiosa *is caused by *Wolbachia *[23]. It is possible that *Wolbachia *or other reproductive parasites are widespread in this genus, causing asexuality in all asexual species. *Wolbachia *strain diversity and abundance in *Bryobia *has not been studied so far.

The above arguments suggest that multiple origins of asexuality are likely. At least seven independent origins of asexuality are required based on our current phylogenetic information (Figure 5). Radiation of asexuals within particular clades remains a possibility, especially because sexual species seem rare within the genus. The general thought that asexuals are always single lineages branching off from closely related sexual relatives is certainly not valid within *Bryobia*. Asexuality seems evolutionary successful, at least in the short term. Asexuality in *Bryobia *is functionally apomictic, resulting in the maintenance of heterozygosity [23]. This may contribute to the success of asexual *Bryobia *species, as high levels of heterozygosity are assumed to be advantageous (heterosis or overdominance; [70]). In other haplodiploid systems parthenogenesis leads to complete homozygosity [71-73].

Additionally, bacterial parasites can play a role in the adaptive success of asexuals. *Wolbachia *has been found in six *Bryobia *species so far [23]. Generally, asexual clones are considered genetically identical if they are identical at their own genomic DNA. However, such genetically identical clones may harbor different bacterial strains. Differences in bacterial composition can influence the fitness of clones, as bacterial symbionts may play a role in protection against parasitoid attack [74] or against fungal pathogens [75]. These differences, and also changes in bacterial composition through occasional horizontal transfers, could play a role in the adaptive success of asexual *Bryobia *species, and of asexuals in general. Furthermore, bacterial genes can be transferred to the host DNA. Recently, Dunning Hotopp et al. [76] showed that an almost complete *Wolbachia *genome was transferred to genome of its host, *Drosophila ananassae*. Other arthropod and nematode species also contained fragments of *Wolbachia *DNA in their genome, indicating that lateral gene transfers occur more often [76]. If occurring in asexuals, such transfers undermine the strict clonality of these asexuals and may play a crucial role in their long term evolutionary success.

## Conclusion

Asexuality is widespread in the genus *Bryobia*. The hypothesis that asexuality originated multiple times is the most plausible, given the fact that reversals to sexuality are unlikely and that more sexual species might be found. A likely explanation is that *Wolbachia*, which causes asexuality in at least two *Bryobia *species [23], has independently infected different *Bryobia *species. The high prevalence of asexual species is in contradiction with the general idea that asexuals are single lineages among sexual sister species. Clonal diversity within the asexual species *B. kissophila *is very high at the mitochondrial DNA. Several distinct clades are found that are paraphyletic to *B. praetiosa*. Past hybridization events and an elevated fixation rate of mutations are possible causes for this high clonal diversity. Reproductive parasites like *Wolbachia *can influence these processes. Moreover, such parasites may play an important role in the evolutionary success of asexuals.

## Authors' contributions

VIDR carried out the molecular genetic studies, performed the analyses, interpreted the data, and drafted the manuscript. VIDR and JAJB collaboratively designed the study and performed the sampling work. JAJB and SBJM participated in the data interpretation and revised the manuscript. All authors read and approved the final manuscript.

## Supplementary Material

Additional file 1 **Details of *Bryobia *samples**. List of *Bryobia *and *Petrobia *samples (excluding *B. kissophila*, see Additional file 2 for details of *B. kissophila *samples). Listed are sample code, species name, sample location (country and locality), host plant, collection date, and GenBank accession numbers (including identical numbers for identical haplotypes, see Figure 3 and 4. Numbers of samples submitted to GenBank are depicted in italics). 'COI' and '28S' indicate the number of individuals sequenced. Click here for file

Additional file 2 **Details of *B. kissophila *samples**. List of *B. kissophila *samples. All samples were collected from *Hedera helix*. 'Clade' lists the clade annotations (A-D) concordant with Figure 4. Listed are sample code, location (country and locality), collection date, and GenBank accession numbers (including identical numbers for identical haplotypes, see Figure 3 and 4. Numbers of samples submitted to GenBank are depicted in italics). 'COI' and '28S' indicate the number of individuals sequenced. Click here for file
