# Supplementary material for: Origins of asexuality in Bryobia mites (Acari: Tetranychidae)
Source: BMC Evol Biol. 2008 May 19;8:153. doi: 10.1186/1471-2148-8-153 (PMC2426695; doi:10.1186/1471-2148-8-153)
Supplement: Additional file 1 — Details of Bryobia samples. List of Bryobia and Petrobia samples (excluding B. kissophila, see Additional file 2 for details of B. kissophila samples). Listed are sample code, species name, sample location (country and locality), host plant, collection date, and GenBank accession numbers (including identical numbers for identical haplotypes, see Figure 3 and 4. Numbers of samples submitted to GenBank are depicted in italics). 'COI' and '28S' indicate the number of individuals sequenced. [file 1471-2148-8-153-S1.pdf]

Additional file 1

Code	Species	Country	Locality	Host plant		Collection date	COI	28S	GenBank accession nr.	
				Common name	Scientific name				COI	28S
ITA8	*B. praetiosa*	Italy	Palestrina	Field Marigold	*Calendula arvensis*	19-Mar-05	1		*EU487089*	
NL10	*B. praetiosa*	Netherlands	Amsterdam	Grass	*Holcus lanatus*	24-May-00	2	2	*EU487091*	EU487059
									EU487088	
NL11	*B. praetiosa*	Netherlands	Enkhuizen	Grass	*Holcus lanatus*	May-00	1		EU487088	
NL12	*B. praetiosa*	Netherlands	Amsterdam	Grass and herbs	-	May-04	1	1	*EU487088*	*EU487059*
NL13	*B. praetiosa*	Netherlands	Utrecht	Trefoil	*Lotus* spec.	17-May-05	1	1	*EU487090*	EU487059
BEL4	*B.* spec. I	Belgium	Olloy-sur-Viroin	Vetches	*Vicia* spec.	26-May-04	1	1	*EU487115*	*EU487050*
NL14	*B.* spec. I	Netherlands	Nieuweschans	Vetches	*Vicia* spec.	23-Jul-05	1		EU487115	
FR14	*B. rubriocules*	France	Aubrives	Apple	*Malus* spec.	26-May-04	2	1	EU487110	*EU487061*
FR15	*B. rubriocules*	France	Peumerit	Apple	*Malus* spec.	11-Aug-06	1		EU487110	
GER4	*B. rubriocules*	Germany	Darmstadt	Apple	*Malus* spec.	25-Jun-04	1	1	*EU487110*	EU487061
GR5	*B. rubriocules*	Greece	Thessaloniki	Damson Plum	*Prunus insititia*	02-Jun-05	2	1	EU487108	*EU487062*
GR6	*B. rubriocules*	Greece	Paliokastro	Sour Cherry	*Prunus cerasus*	04-Jun-05	1		EU487108	
GR7	*B. rubriocules*	Greece	Paliokastro	Blackthorn	*Prunus spinosa*	04-Jun-05	1		EU487109	
GR8	*B. rubriocules*	Greece	Paliokastro	Plum	*Prunus domestica*	4-Jun-05		1		EU487062
GR12	*B. rubriocules*	Greece	Panorama	Almond	*Amygdalus webbii*	06-Jun-05	1	1	*EU487112*	EU487061
NL15	*B. rubriocules*	Netherlands	Amsterdam	Apple	*Malus* spec.	17-May-04	1		EU487110	
NL16	*B. rubriocules*	Netherlands	Amsterdam	Plum	*Prunus* spec.	10-May-06	1	1	*EU487108*	*EU487063*
PL1	*B. rubriocules*	Poland	Przenosza	Apple	*Malus* spec.	13-Aug-05	1		EU487110	
PL2	*B. rubriocules*	Poland	Skrudzina	Apple	*Malus* spec.	14-Aug-05	1		EU487110	
PL3	*B. rubriocules*	Poland	Kwaczala	Apple	*Malus* spec.	21-Aug-05	1		EU487110	
PL4	*B. rubriocules*	Poland	Makow Podhalanski	Apple	*Malus* spec.	21-Aug-05	1		EU487111	
SLK1	*B. rubriocules*	Slovakia	Piesok	-	*Prunus* spec.	20-May-04	1	1	*EU487109*	EU487061
SLK3	*B. rubriocules*	Slovakia	Casta	Apple	*Malus* spec.	21-May-04	1		*EU487111*	
SLK4	*B. rubriocules*	Slovakia	Piesok	Apple	*Malus* spec.	23-May-04	1		EU487110	
BEL5	*B. sarothamni*	Belgium	Vierves sur Viroin	Common Broom	*Cytisus scoparius*	26-May-04	1	1	*EU487124*	*EU487056*
BEL6	*B. sarothamni*	Belgium	Vierves sur Viroin	Common Broom	*Cytisus scoparius*	26-May-04	1	1	EU487124	EU487056
FR16	*B. sarothamni*	France	Vireux	Common Broom	*Cytisus scoparius*	26-May-04	1	1	*EU487125*	EU487056
FR20	*B. sarothamni*	France	Peumerit	Common Broom	*Cytisus scoparius*	11-Aug-06	1	1	*EU487123*	EU487056
FR21	*B. sarothamni*	France	Piriac sur mer	Common Broom	*Cytisus scoparius*	11-Aug-06	1	1	EU487125	EU487056
NL17	*B. sarothamni*	Netherlands	Schoorl	Common Broom	*Cytisus scoparius*	27-Jun-04	1		EU487124	
FR17	*B. berlesei*	France	Vireux	Common Broom	*Cytisus scoparius*	26-May-04	3	1	*EU487094*	*EU487057*
ITA9	*B. berlesei*	Italy	Sabaudia	Common Broom	*Cytisus scoparius*	21-Mar-05	1	1	*EU487093*	EU487057
NL18	*B. berlesei*	Netherlands	Hilversum	Common Broom	*Cytisus scoparius*	12-May-04	1		EU487093	
SP7	*B. berlesei*	Spain	El Rocio	Broom spec.	? (Fabaceae)	15-Apr-05	1		*EU487095*	
FR19	*B.* spec. II	France	St. Maximine	Broom spec.	? (Fabaceae; Genisteae)	17-May-05	1		*EU487100*	
GR9	*B.* spec. II	Greece	Chortiatis	Broom spec.	? (Fabaceae; Genisteae)	06-Jun-05	1		*EU487101*	
SP8	*B.* spec. II	Spain	Nulles	Broom spec.	? (Fabaceae; Genisteae)	14-Apr-04	1		EU487100	

Additional file 1 - *continued*

Code	Species	Country	Locality	Host plant		Collection date	COI	28S	GenBank accession nr.	
				Common name	Scientific name				COI	28S
SP9	*B*. spec. III	Spain	Olesa de Bonesvalls	Gorse	*Ulex* spec.	14-Apr-04	1		*EU487097*	
SP10	*B*. spec. III	Spain	Villa rodona	Gorse	*Ulex* spec.	14-Apr-04	1		*EU487096*	
SP11	*B*. spec. III	Spain	Castello, vill. d'Escornalbou	Gorse	*Ulex* spec.	15-Apr-04	1	1	*EU487098*	*EU487058*
SP12	*B*. spec. III	Spain	Ribesalbes	Gorse	*Ulex* spec.	19-Apr-04	1		*EU487099*	
SP13	*B*. spec. III	Spain	Olivella	Gorse	*Ulex* spec.	20-Apr-04	1		EU487097	
POR4	*B*. spec. IV	Portugal	Caldas de Monchique	Mallow	*Malva* spec.	01-Feb-05	2	1	*EU487106*<br>*EU487107*	*EU487065*
ITA11	*B*.spec. V	Italy	S. Felice Circeo	Grass and herbs	-	21-Mar-05	1	1	*EU487113*	EU487049
POR7	*B*.spec. V	Portugal	Alferce	Pitch Trefoil	*Psoralea bituminosa*	01-Feb-05	1	1	*EU487114*	*EU487049*
SP17	*B*.spec. V	Spain	Villa rodona	Vetches	*Vicia* spec.	14-Apr-04	1		EU487113	
SP18	*B*.spec. VI	Spain	Trujillanos	Broom spec.	? (Fabaceae; Genisteae)	27-Apr-05	1	1	*EU487102*	*EU487055*
SP19	*B*.spec. VI	Spain	Alhaurin el Grande	Broom spec.	? (Fabaceae; Genisteae)	29-Apr-05	1	1	*EU487103*	EU487055
FR18	*B*.spec. VII	France	Le Clerjus	Grass	*Holcus* spec.	24-Jul-05	1	1	*EU487104*	EU487064
NL20	*B*.spec. VII	Netherlands	Hilversum	Grass	*Holcus lanatus*	12-May-04	1	1	*EU487105*	*EU487064*
ITA10	*P. tunisea*	Italy	S. Felice Circeo	Brome	*Anisantha* spec.	21-Mar-05	1		EU487118	
SP14	*P. tunisea*	Spain	Begues	Grass	-	14-Apr-04	1		*EU487119*	
SP15	*P. tunisea*	Spain	Olesa de Bonesvalls	Grass	-	14-Apr-04	2		*EU487118*	
SP16	*P. tunisea*	Spain	Marmellar	Grass	-	14-Apr-04	1	1	EU487119	*EU487052*
POR5	*P*. spec. I	Portugal	Olhao	Soursob	*Oxalis pes-caprae*	07-Feb-05	1		EU487122	
POR6	*P*. spec. I	Portugal	Caldas de Monchique	Soursob	*Oxalis pes-caprae*	01-Feb-05	1	1	*EU487122*	*EU487051*
CH1	*P. harti*	China	Huanyaguan	Soursob	*Oxalis pes-caprae*	03-Aug-05	1	1	*EU487121*	EU487054
CH2	*P. harti*	China	Dali	Soursob	*Oxalis pes-caprae*	13-Aug-05	1		EU487121	
GR10	*P. harti*	Greece	Thessaloniki	Soursob	*Oxalis pes-caprae*	02-Jun-05	1		EU487120	
GR11	*P. harti*	Greece	Pella	Soursob	*Oxalis pes-caprae*	31-May-05	1	1	*EU487120*	*EU487054*
NL19	*P*. spec. II	Netherlands	Utrecht	Trefoil	*Lotus* spec.	17-May-05	1	1	*EU487116*	*EU487053*
POR8	*P*. spec. II	Portugal	Alferce	Pitch Trefoil	*Psoralea bituminosa*	01-Feb-05	1		*EU487117*	
