# Supplementary material for: Origins of asexuality in Bryobia mites (Acari: Tetranychidae)
Source: BMC Evol Biol. 2008 May 19;8:153. doi: 10.1186/1471-2148-8-153 (PMC2426695; doi:10.1186/1471-2148-8-153)
Supplement: Additional file 2 — Details of B. kissophila samples. List of B. kissophila samples. All samples were collected from Hedera helix. 'Clade' lists the clade annotations (A-D) concordant with Figure 4. Listed are sample code, location (country and locality), collection date, and GenBank accession numbers (including identical numbers for identical haplotypes, see Figure 3 and 4. Numbers of samples submitted to GenBank are depicted in italics). 'COI' and '28S' indicate the number of individuals sequenced. [file 1471-2148-8-153-S2.pdf]

Additional file 2

Clade	Code	Country	Locality	Collection date	COI	28S	GenBank accession nr.	
							COI	28S
A	FR13	France	Vireux	26-May-04	3	1	*EU487092*	EU487060
	NL9	Netherlands	Nes (Ameland)	9-Apr-04	2	1	EU487092	EU487060
	NL21	Netherlands	Nes (Ameland)	9-Apr-04	1		EU487092	
B	BEL1	Belgium	Lompret	26-May-04	1		EU487067	
	BEL2	Belgium	Olloy-sur-Viroin	26-May-04	1		EU487067	
	BEL3	Belgium	Werpin	23-Apr-06	1		EU487067	
	DEN1	Denmark	Vester Aby	3-May-05	1		EU487076	
	DEN2	Denmark	Varde	4-May-05	1		EU487067	
	ENG1	England	Edale	21-Aug-04	1		*EU487073*	
	ENG2	England	Shrewsbury	28-Aug-04	1		EU487067	
	ENG3	England	Little Stretton	1-Sep-04	1		EU487067	
	FR1	France	Paris	25-Apr-04	1		EU487067	
	FR2	France	Angers	10-May-04	1	1	*EU487067*	*EU487060*
	FR3	France	Givet	26-May-04	1		EU487067	
	FR4	France	Piriac sur Mer	27-Jul-04	1		EU487067	
	FR5	France	Kermoizan	Jul-04	1		EU487067	
	FR6	France	Peymeinade	15-May-05	1		EU487067	
	FR7	France	Villeneuve sur Avignon	18-May-05	1		EU487067	
	FR8	France	Liverdum	24-May-05	1		EU487067	
	FR9	France	St Clement de Riviere	26-Jun-04	1		*EU487068*	
	FR10	France	Bellegarde	23-May-05	1		*EU487071*	
	FR11	France	Gonfaron	16-May-05	1		*EU487077*	
	FR12	France	Brignolle	17-May-05	1		EU487077	
	GER1	Germany	Mainz	Apr-04	1		EU487067	
	GER2	Germany	Darmstadt	25-Jun-04	1		EU487067	
	GER3	Germany	Erfurt	5-Feb-05	1		EU487067	
	GR1	Greece	Thessaloniki	2-Jun-05	1		EU487081	
	GR2	Greece	Thermi	27-May-05	1		EU487081	
	GR3	Greece	Athene	29-May-05	1		EU487081	
	GR4	Greece	Thessaloniki	2-Jun-05	1		*EU487081*	
	ITA1	Italy	Palestrina	19-Mar-05	1		*EU487076*	
	ITA2	Italy	Fiuggi	19-Mar-05	1		EU487067	
	ITA3	Italy	Pompei	20-Mar-05	1		EU487067	
	ITA4	Italy	S. Felice Circeo	21-Mar-05	1		EU487067	
	ITA5	Italy	Rome	22-Mar-05	1		EU487067	
	ITA6	Italy	Certosa di Trisulti	19-Mar-05	2		*EU487070*	
	ITA7	Italy	Sabaudia	21-Mar-05	1		*EU487075*	
	NL1	Netherlands	Hardegarijp	6-Apr-04	1	1	*EU487079*	EU487060
	NL2	Netherlands	Nijmegen	4-May-04	1		*EU487069*	
	NL3	Netherlands	Amsterdam	13-Apr-04	1	1	*EU487078*	EU487060
	NL4	Netherlands	Den Burg (Texel)	15-Feb-04	1		EU487078	
	NL5	Netherlands	West-Terschelling	2-Apr-05	2		EU487078	
	NL6	Netherlands	Valkenburg	4-May-04	1		EU487074	
	NL7	Netherlands	Varsseveld	15-May-04	1		EU487076	
	NL8	Netherlands	Utrecht	25-Apr-04	1		EU487076	
	POR2	Portugal	Quinta da Balaia	5-Feb-05	1		*EU487074*	
	SA1	South Africa	Johannesburg	13-Aug-05	1	1	*EU487080*	EU487060
	SLK2	Slovakia	Modra	23-May-04	1		*EU487072*	
	SLV1	Slovenia	Maribor	10-May-05	1		*EU487066*	
	SWE1	Sweden	Lund	3-Apr-05	1		EU487067	
	SWE2	Sweden	Lund	3-Apr-05	1		EU487067	
	SWE3	Sweden	Lund	3-Apr-05	1		EU487067	
C	US1	United Sates	Riverside	24-May-05	1	1	*EU487087*	EU487060
	US2	United Sates	Newark	17-May-05	1		EU487087	
D	POR1	Portugal	Portimao	2-Feb-05	1		*EU487083*	
	POR3	Portugal	Quinta da Balaia	5-Feb-05	1		*EU487084*	
	SP1	Spain	Begues	14-Apr-04	2		*EU487085*	
	SP2	Spain	Castello, vill. d'Escornalbou	15-Apr-04	1	1	*EU487082*	EU487060
	SP3	Spain	Gatova	19-Apr-04	1	1	*EU487086*	EU487060
	SP4	Spain	Alcossebres	19-Apr-04	1	1	EU487082	EU487060
	SP5	Spain	Alcossebres	20-Apr-04	1		EU487082	
